# A generalized birth and death process for modeling the fates of gene duplication

**DOI:** 10.1186/s12862-015-0539-2

**Published:** 2015-12-08

**Authors:** Jing Zhao, Ashley I. Teufel, David A. Liberles, Liang Liu

**Affiliations:** Department of Statistics, University of Georgia, 101 Cedar Street, Athens, GA 30602 USA; Department of Molecular Biology, University of Wyoming, Laramie, WY 82071 USA; Center for Computational Genetics and Genomics and Department of Biology, Temple University, Philadelphia, PA 19122 USA; Institute of Bioinformatics, University of Georgia, Athens, GA 30602 USA

**Keywords:** Gene duplication, Phylogenetic methods, Probabilistic models, Birth-death processes, Stochastic processes

## Abstract

**Background:**

Accurately estimating the timing and mode of gene duplications along the evolutionary history of species can provide invaluable information about underlying mechanisms by which the genomes of organisms evolved and the genes with novel functions arose. Mechanistic models have previously been introduced that allow for probabilistic inference of the evolutionary mechanism for duplicate gene retention based upon the average rate of loss over time of the duplicate. However, there is currently no probabilistic model embedded in a birth-death modeling framework that can take into account the effects of different evolutionary mechanisms of gene retention when analyzing gene family data.

**Results:**

In this study, we describe a generalized birth-death process for modeling the fates of gene duplication. Use of mechanistic models in a phylogenetic framework requires an age-dependent birth-death process. Starting with a single population corresponding to the lineage of a phylogenetic tree and with an assumption of a clock that starts ticking for each duplicate at its birth, an age-dependent birth-death process is developed by extending the results from the time-dependent birth-death process. The implementation of such models in a full phylogenetic framework is expected to enable large scale probabilistic analysis of duplicates in comparative genomic studies.

**Conclusions:**

We develop an age-dependent birth-death model for understanding the mechanisms of gene retention, which allows a gene loss rate dependent on each duplication event. Simulation results indicate that different mechanisms of gene retentions produce distinct likelihood functions, which can be used with genomic data to quantitatively distinguish those mechanisms.

## Background

A gene family is a group of genes with similar sequences that show evidence of descent from a common ancestor [1–3]. This includes orthologs that originate through speciation as well as duplicates (modeled here) that can be found within a species or shared between species from an older duplication event that predated speciation. The large number of genes per family suggests that the newly arisen gene duplicates are potentially major contributors to evolutionary novelties [4–7]. Gene duplication can provide raw genetic material for evolutionary forces to act on. Although a majority of duplicate genes may be silenced by degenerative mutations or lost due to population dynamics, some duplicated genes are able to evolve novel functions permanently preserved in the population [8, 9]. Accurately estimating the timing and mode of gene duplications along the evolutionary history of species can provide invaluable information about underlying mechanisms by which the genomes of organisms evolved and the genes with novel functions arose [10].

Several biological models have been proposed to depict the mechanisms that lead to different evolutionary fates of a gene duplicate [11–14]. Nonfunctionalization refers to the process in which mutations occur on one of the gene duplicates and produce a non-functional protein [11, 15]. The neofunctionalization model [16] assumes that duplication itself does not affect fitness. Although a duplicate is most likely to be pseudogenized by degenerative mutation (nonfunctionalization) or lost due to population dynamics [9], the redundant copy may occasionally acquire a new beneficial function through mutation that will be preferentially preserved in the population. While this function may subsequently be optimized and accommodated within the genome structure (assuming a coding sequence change) by an evolutionary Stokes shift [17], the initial event leading to retention is a single beneficial change. The waiting time for this single change gives rise to a convexly decaying hazard function when modeled together with non-functionalizing changes and is referred to as the neofunctionalization model (see [15, 18, 19] for a review). The duplication-degeneration-complementation model [20] describes a so-called subfunctionalization mechanism in which two gene copies are partially damaged by degenerated mutations. Both copies must be maintained in order to perform the original function of the gene [21, 22]. This model, called subfunctionalization, involves a waiting time for multiple events to occur as deleterious substitutions accumulate in both copies before the retaining mutation can occur. This waiting time for multiple changes gives rise to a switch from a convex to a concave (sigmoidal) hazard function when modeled together with non-functionalizing mutations (again, see [15, 18, 19] for a review and engaged discussion). In addition to the processes acting on individual genes, large-scale gene duplication events (for example, whole genome duplication) may have occurred and produced multiple interacting genes together creating an additional retention mechanism. Dosage balance promotes the retention of duplicated interaction networks, as loss of interaction stoichiometry can lead to declines in fitness. This gives rise to very different retention dynamics compared to neofunctionalization or subfunctionalization (see [15, 18, 19] for a review). The mechanistic models described for nonfunctionalization, subfunctionalization, neofunctionalization, and dosage balance represent one of many conceivable modeling frameworks for duplicate gene retention (see [19] for an enhanced discussion). The models here are used within a single population, reflecting a lineage of a phylogenetic tree, but the ultimate aim is to extend their use into an interspecific phylogenetic framework with the population genetic assumptions that accompany this. Simpler models have already been incorporated into a fuller phylogenetic framework of this nature (see for example [23]).

Accurately reconstructing the evolution of gene families requires informative datasets, powerful mathematical models, and efficient computational algorithms. Advanced biotechnologies provide a vast amount of genetic data for understanding the evolution of gene families [24, 25]. Meanwhile, probabilistic models, describing the process of gene family evolution, significantly enhance our ability to extract useful information from genetic data [26–29]. The birth-death (BD) model [30], which has been broadly applied in analyzing species phylogenies [25, 29, 31, 32], could also be adopted in phylogenetic analysis of gene families [33]. In 1975, Thompson [34] introduced a phylogenetic model based on the birth-death process to understand the evolution of human populations. Under the generalized birth-death model, Nee et al. [35] derived a reconstructed evolutionary process [36] to estimate birth and death rates in a interspecific phylogenetic framework. Rannala and Yang [37] developed a birth-death phylogenetic model for estimating phylogenetic trees from molecular sequence data. Aldous and Popovic [38] proposed a continuous-time critical branching process conditioned on the number of species in the present, with the assumption that the birth and death rates are identical in macroevolution, which was later relaxed by Gernhard [39, 40] to allow uncorrelated birth and death rates. With the assumption of constant birth and death rates, Stadler [41] derived the probability density function of a phylogenetic tree under the birth-death model. Recently, time-dependent BD processes have attracted more attention as a mode of performing hypothesis-driven research [42–45]. Rabosky [42] distinguished rate-variable models of diversification from rate-constant models by fitting BD models using likelihood methods. Hohna [44, 46] and Hallinan [45] studied the reconstructed process with time-dependent rates in a more general setting by relaxing the assumptions about the number of species and the time of the process. The BD model was first adopted in [47] and further extended by other researchers to reconcile gene and species trees (Arvestad et al. [48], Akerborg et al. [23], Rasmussen and Kellis [49] and Sjostrand et al. [50]). Recently, Boussau et al. [51] established a BD phylogenetic model for co-estimating gene and species trees without the need of estimation of divergence times in species trees and duplication and loss rates.

The current computational methods for analyzing gene family data (including gene duplication and loss) suffer a variety of weakness that need to be addressed. There is no probabilistic model embedded in a birth-death phylogenetic modeling framework that can take into account the effects of different evolutionary mechanisms of gene retention when analyzing gene family data. It is desirable to build a stochastic model as a good approximation to the real biological process of gene duplication and loss. Such probabilistic models can both add biological realism to improve the fit of the model to the data as well as enable mechanistic inference that is currently not possible. In this study, we integrate several evolutionary mechanisms of gene retention into the age-dependent BD model [42–45], in which the loss rate is a function of the ages of gene copies. Moreover, we derive the probability density function of gene duplication times for each mechanism. The conditional density function of a duplication time given the previous duplication time is derived from the reconstructed process under the generalized birth-death model [35, 52]. The conditional density function can be utilized to calculate the joint density of duplication times, and to efficiently simulate duplication times under the generalized BD model. The simulation results suggest that the maximum likelihood approach can accurately estimate the parameters in the generalized BD model for different mechanisms of gene retention, and the proposed gene-retention model can be used to detect the underlying mechanism that drives the evolutionary process of duplicates within a gene family.

## Methods

### Modeling the loss rate

For simplicity, we consider the process of gene duplication/loss in a single population. For a single population, we assume that a gene copy may duplicate or die at time *t*. The homogeneous birth-death model assumes that the rate of loss (hazard) of a duplicated gene is constant through time [11, 53]. This expectation is consistent with the nonfunctionalization process, but does not take into account any of the processes of neofunctionalization and subfunctionalization, which can affect the loss rate of gene duplicates. The birth-death model for the fates of gene retention (nonfunctionalization, subfunctionalization, neofunctionalization, and dosage balance) includes a time-dependent loss rate and a constant duplication rate λ. The time-dependent loss rates will be extended to age-dependent loss rates in the age-dependent birth-death model (see section 2.3). The process starts at time 0, and the number of gene copies at time 0 is 2. The process of gene duplication and loss occurs under the following postulates [54]: (1) the probability that a duplication will occur during an infinitesimal interval (t, t + Δt] is *n*_*t*_λΔ*t* + o(Δt), while the probability that no duplication will occur is 1- *n*_*t*_λΔt + o(Δt), and (2) the probability that a gene duplicate will be lost during an infinitesimal interval (t, t + Δt] is *n*_*t*_*μ*_*t*_Δt + o(Δt), while the probability that no loss will occur is 1- *n*_*t*_*μ*_*t*_ Δt + o(Δt), in which the loss rate *μ*_*t*_ is a function of time t.

We introduce three formulas for the loss rate *μ*_*t*_ based on the processes of nonfunctionalization, neofunctionalization, and subfunctionalization, with assumptions about these processes made in the introduction and also described in [45]. For nonfunctionalization, the loss rate *μ*_*t*_ is constant over time *t*, i.e., *μ*_*t*_ = *μ*. The neofunctionalization hazard rate (instantaneous rate of duplicate copy loss) declines with time [55]. Averaging across the probability of hitting a neofunctionalizing substitution, the nonfunctionalization probability for duplicate genes declines, leading to the overall decline of duplicate loss over long evolutionary time periods [19]. This convexly declining loss rate has been described with a Weibull hazard function to characterize the average process (the process for a single gene with a known neofunctionalization event would be a discrete jump in the hazard rate) [18]. We use an exponential function to model the loss rate of neofunctionlization, i.e., *μ*_*t*_ = *αe*^− *tα*^ for 0 < *α* < 1. Further, the subfunctionalization loss rate behavior has been characterized to be concavely (sigmoidally) declining based upon theoretical expectations of a waiting time for complementary mutations [18, 20]. Konrad [15] introduced an extended exponential hazard function to describe the instantaneous rate of loss. We adopt a generalized logistic function for the loss rate *μ*_*t*_ of subfunctionalization, i.e., ${\mu}_t=\frac{\alpha {e}^{\gamma -t}}{1+{e}^{\gamma -t}}$, in which the scale parameter 0 < *α* < 1 and known location parameter γ > 0.

### The time-dependent birth-death model

We are interested in the probability distribution of duplication times of the reconstructed lineages (the lineages that have survived to the present time), because the phylogeny reconstructed from the sequences of contemporary species does not include the extinct lineages [35]. The pure birth process of the reconstructed lineages can be derived from a generalized birth-death process [34, 36]. We use the following notations which are defined closely to Nee et al. [35] throughout this paper. Let *t*_2_ = 0 be the first duplication time at the root of the tree, and *T* be the present time (we are looking forward in time, i.e., *T* > 0). Let *n*_*T*_ be the number of lineages at the present time *T*. Let *n*_*i*_ be the number of reconstructed lineages alive at *t*_*i*_ that survive to the present. We use {*t*_*i*_ | *i* = 2, …, *n*_*T*_} to denote the duplication times of *n*_*T*_ lineages at the tips of a phylogenetic tree, and *t*_*2*_ *< t*_*3*_ *< t*_*4*_ *< … < T*. Let P(*τ, T*) be the probability that one lineage at time *τ* leaves multiple descendants at the present time *T*, i.e., P(*τ, T*) = *P*(*n*_*T*_ >0 | *n*_*τ*_ =1) [34–36, 44],

1 $$ P\left(\tau, T\right)={\left[1+{\displaystyle {\int}_{\tau}^T{\mu}_t{e}^{\rho \left(\tau, t\right)}dt}\right]}^{-1}. $$

In Eq. (1), *ρ*(*τ*, *T*) = ∫_*τ*_^*T*^(*μ*_*s*_ − *λ*)*ds*. Since the integral ∫_*τ*_^*T*^*μ*_*t*_*e*^*ρ*(*τ*,*t*)^ in Eq. (1) is analytically intractable, it is approximated by a Monte Carlo method. We define *u*_*ij*_ as the probability *P*(*n*_*j*_ > 1 | *n*_*i*_ = 1) that one lineage at time *t*_*i*_ leaves multiple descendant reconstructed lineages at a later time *t*_*j*_. This probability has been derived under the time-dependent BD model, i.e., ${u}_{ij}=P\left({n}_j>1\Big|{n}_i=1\right)=1-P\left({t}_i,{t}_j\right){e}^{\rho \left({t}_i,{t}_j\right)}$ (see Eq. (8) in [45]). Given the number *n*_*T*_ of lineages at the present time *T* and the number *n*_0_ of lineages at time 0, the probability density function of the duplication times *t* = {*t*_*i*_ | *i* = *n*_*0*_ + 1, …, *n*_*T*_} is given by [45]

2 $$ f\left(t\Big|{n}_T,{n}_0,T\right)=\frac{{\displaystyle {\prod}_{i={n}_0+1}^{n_T}}\left(i-1\right)\lambda P\left({t}_i,T\right){\left(1-{\eta}_{t_{i-1},{t}_i}\right)}^{i-1}}{\left(\begin{array}{c}\hfill {n}_T-1\hfill \\ {}\hfill {n}_0-1\hfill \end{array}\right){\left(1-{\eta}_{0,T}\right)}^{n_0}{\eta}_{0,T}^{n_T-{n}_0}}. $$

In (2), ${\eta}_{ij}=1-\frac{1-{u}_{iT}}{1-{u}_{jT}}$. The conditional probability distribution of duplication time *t*_*i*_ (*i* > 2), given its previous duplication time *t*_*i-1*_, *T* and *n*_*T*_, is given by [45]

3 $$ f\left({t}_i\Big|{t}_{i-1},{n}_T,T\right)=\frac{f\left({t}_i\Big|{t}_{i-1}\right)P\left({n}_T\Big|{n}_{t_i},T\right)}{P\left({n}_T\Big|{n}_{t_{i-1}},T\right)}. $$

In Eq. (3), $f\left({t}_i\Big|{t}_{i-1}\right)=\left(i-1\right)\lambda P\left({t}_i,T\right){\left(1-{\eta}_{t_{i-1},{t}_i}\right)}^{i-1}$ (see Eq. (19) and (23) in [45]). With the conditional densities *f*(*t*_*i*_|*t*_*i* − 1_, *n*_*T*_, *T*) of duplication times, the duplication events between times 0 and *T* can be simulated recursively in forward direction. The conditional density in (3) differs from the density of duplication times derived by Hohna [44], in which the duplication events are treated as a random sample from a common probability distribution.

### The age-dependent birth-death model

The time-dependent birth-death model described in the previous section starts with a single population corresponding to the lineage of a phylogenetic tree and assumes a molecular clock that starts ticking for all duplicates at the root. Thus, in the time-dependent birth-death model, the loss rate *μ*_*t*_ of a gene copy is a function of time *t*. However, the loss rate μ_t_ should be a function of the ages of gene copies. In this section, the time-dependent birth-death process is extended to the age-dependent process, where the clock for each duplicate starts ticking at its birth. When the loss rate is constant (i.e., nonfuncitonalization), the age-dependent model is identical with the time-dependent model. Thus, we only describe the age-dependent model for neofunctionalizaiton and subfunctionalization. In the age-dependent model, the expressions for the loss rates of neofunctionalization and subfunctionalization remain unchanged (see section Modeling the loss rate), except that time *t* is replaced with the age *t’* of the gene copy, i.e., ${\mu}_{t^{\hbox{'}}}=\alpha {e}^{-{t}^{\hbox{'}}\alpha }$ for neofunctionalization and ${\mu}_{t^{\hbox{'}}}=\frac{\alpha {e}^{\gamma -{t}^{\hbox{'}}}}{1+{e}^{\gamma -{t}^{\hbox{'}}}}$ for subfunctionalization. Moreover, it is assumed that the number of gene copies increases or decreases by 1 or remains the same during an infinitesimal interval (t, t + Δt] with probabilities described in (4a-c)

4a $$ P\left({n}_{t+\Delta t}={n}_t+1\right)={n}_t\lambda \Delta t+o\left(\Delta \mathrm{t}\right) $$

4b $$ P\left({n}_{t+\Delta t}={n}_t-1\right)={\displaystyle \sum_{i=1}^{n_t}}{\mu}_{t_i^{\hbox{'}}}\Delta t+o\left(\Delta \mathrm{t}\right) $$

4c $$ P\left({n}_{t+\Delta t}={n}_t\right)=1-\left({n}_t\lambda +{\displaystyle \sum_{i=1}^{n_t}}{\mu}_{t_i^{\hbox{'}}}\right)\Delta t+o\left(\Delta \mathrm{t}\right) $$

In (4b), ${\mu}_{t_i^{\hbox{'}}}$ is the loss rate of gene copy *i* at the age of *t*_*i*_^'^ for *i* = 1, 2, …, *n*_*t*_. Let *t*_*i*_^0^ be the duplication time of gene copy *i*. The age *t*_*i*_^'^ of gene copy *i* is a random variable, because it is a function of the random duplication time *t*_*i*_^0^, i.e., *t*_*i*_^'^ = *t* − *t*_*i*_^0^. Therefore, (4b) and (4c) are integrated over all possible values of ${\mu}_{t_i^{\hbox{'}}}$ with respect to the probability density function *f*(*t* ') of the age *t* ' of a gene copy. The age-dependent loss rate ${\mu}_{t_i^{\hbox{'}}}$ in (4b) and (4c) is replaced with its expectation $E\left({\mu}_{t_i^{\hbox{'}}}\right)$. Since all *t*_*i*_^'^ s have the same probability distribution, the loss rates of *n*_*t*_ gene copies have the same expected values. Let *t*^0^ be the most recent duplication time of a gene copy that survives to time *t*. Since *t*^0^ is the most recent duplication time, it indicates that no duplication or loss events have occurred between *t*^0^ and *t* on the gene copy. It has been shown that the number of duplication or loss events follows the Poisson distribution with mean ∫_0_^*t*^(*λ* + *μ*_*x*_)*dx*. The probability of no duplication or loss events occurring within the time interval [0, *t*] is equal to ${e}^{-{\displaystyle {\int}_0^t\left(\lambda +{\mu}_x\right)dx}}$. Thus, the probability density of duplication time *t*^0^ is proportional to ${D}_{t^0}{e}^{-{\displaystyle {\int}_0^t\left(\lambda +{\mu}_x\right)dx}}$ for 0 < *t*^0^ < *t*, in which ${D}_{t^0}$ is the duplication rate at time *t*^0^ and ${e}^{-{\displaystyle {\int}_0^t\left(\lambda +{\mu}_x\right)dx}}$ is the probability that *t*^0^ is the most recent duplication time of the gene copy. Given that duplication occurs on a specific lineage, ${D}_{t^0}$ is equal to the duplication rate λ. Thus, the probably density of the most recent duplication time *t*^0^ is

5 $$ f\left({t}^0\right)=\frac{e^{-{\displaystyle {\int}_{t^0}^t}\left(\lambda +{\mu}_x\right)dx}}{{\displaystyle {\int}_0^t}\left({e}^{-{\displaystyle {\int}_{t^0}^t}\left(\lambda +{\mu}_x\right)dx}\right)d{t}^0} $$

Because the gene age *t’* is equal to *t* – *t*^*0*^, the probability density of age *t’* for 0 < *t*^0^ < *t* is given by

6 $$ f\left(t\hbox{'}\right)=\frac{e^{-{\displaystyle {\int}_{t-t\hbox{'}}^t}\left(\lambda +{\mu}_x\right)dx}}{{\displaystyle {\int}_0^t}\left({e}^{-{\displaystyle {\int}_{t-t\hbox{'}}^t}\left(\lambda +{\mu}_x\right)dx}\right)dt\hbox{'}} $$

Since the denominator in (6) is intractable, it is approximated by Monte Carlo simulation. It follows that the mean loss rate at time *t* is ${\phi}_t=E\left({\mu}_{t_i^{\hbox{'}}}\right)={\displaystyle {\int}_0^t{\mu}_{t\hbox{'}}f\left(t\hbox{'}\right)dt\hbox{'}}$. Thus, the postulates in (4b) and (4c) become *P*(*n*_*t* + Δ*t*_ = *n*_*t*_ − 1) = *nϕ*_*t*_Δt + *ο*(Δt) and *P*(*n*_*t* + Δ*t*_ = *n*_*t*_) = 1 − *n*_*t*_(*λ* + *ϕ*_*t*_)Δ*t* + *ο*(Δt). The loss rate in Eq. (1) is replaced by the mean loss rate *ϕ*_*t*_ accordingly and *P*(*τ*, *T*) is modified as

7 $$ P\left(\tau, T\right)={\left[1+{\displaystyle {\int}_{\tau}^T{\phi}_t{e}^{\rho \left(\tau, t\right)}dt}\right]}^{-1} $$

Finally, the joint and conditional probability density of duplication times (in Eq. 2–3) for the age dependent model remain unchanged, except that the loss rate *μ*_*t*_ in Eq. (2–3) is replaced with the mean loss rate *ϕ*_*t*_.

## Results

### Simulation for the time-dependent model

To evaluate the performance of the time-dependent birth-death model on simulated data where the true values of parameters are known, we generated duplication times of gene copies using the rejection-sampling algorithm with the conditional probability density function of duplication times in Eq. (3). We found the maximum likelihood score for the conditional probability distribution using an optimization function *optim* in R. The maximum score was used as the upper bound in the rejection-sampling algorithm. Specifically, duplication times were simulated from Eq. (3) with a fixed current time *T = 10* and a fixed number of gene copies *n*_*T*_ = 32 at time *T*. The first duplication time is set to 0, i.e., *t*_*2*_ = 0; the second one is simulated conditional on the first one and so on so that additional 30 duplication times are generated sequentially*.* Duplication events were generated under each of 3 duplication mechanisms (nonfunctionalization, neofunctionalization, and subfunctionalization) with different parameterizations specified in Table 1. We set a constant duplication rate *λ* = 0.2 for all simulations (Table 1). The loss rates were determined by the equations described previously for nonfunctionalization, neofunctionalization, and subfunctionalization models with parameters shown in Table 1. The values of parameters were selected such that three mechanisms have the same initial deletion rate.

**Table 1:** The values of parameters used in simulating duplication times under nonfunctionalization, neofunctionalization, and subfunctionalization are shown

	λ	μ	α
Nonfunctionalization	0.2	0.8	
Neofunctionalization	0.2		0.8
Subfunctionalization	0.2		0.8

**Fig. 1:**
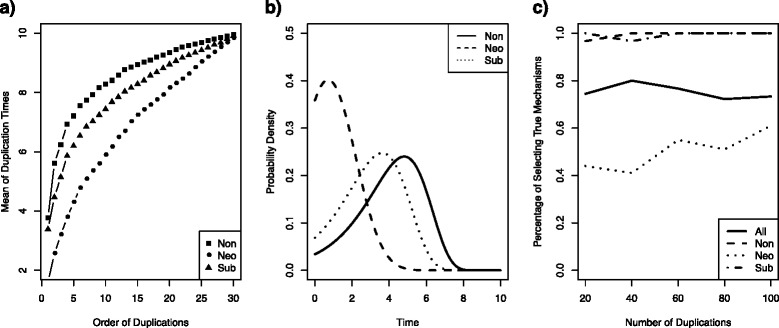
Simulation results of the time-dependent model: (**a**) the means of duplication times simulated with 100 replicates for nonfunctionalization, neofunctionalization, and subfuncitonalization are shown; (**b**) the probability density curves of duplication times for nonfunctionalization, neofunctionalization, and subfunctionalization under the model are shown; (**c**) the percentage of samples identifying the true mechanism with AIC

For each mechanism, simulation was repeated 100 times. The mean of simulated duplication times for each of three mechanisms are shown in Fig. 1a. Duplication times simulated under different mechanisms show distinct patterns. Given the present time *T* and the number of gene copies *n*_*T*_, the overall duplication times for nonfunctionalization tend to be larger than those for neofunctionalization and subfunctionalization, and duplication times for neofunctionalization appear to be smaller than subfunctionalization. The curves of duplication times for nonfunctionalization, neofunctionalization, and subfunctionalization are well separated (Fig. 1a), even though three mechanisms have the same duplication rate and the same starting deletion rate. These results indicate that duplication times can be used to distinguish different mechanisms of gene retention, and to make inference about the underlying mechanism that generated the observed duplication times given the assumptions of the duplication models and their relationship to the underlying biology. These results are consistent with the caveat that the time-dependent process uses a tree-dependent clock rather than the more biological situation of a duplication-event specific process. The extension to the age-dependent birth-death model will be discussed below. The joint probability density function in Eq. (2) can be used to obtain the maximum likelihood estimates (MLE) of parameters in the time-dependent model, when duplication times are given as input data. To visualize the divergence of the probability density functions of three mechanisms, we plotted the density curves of the first duplication time for nonfunctionalization, neofunctionalization, and subfunctionalization (Fig. 1b) with the values of parameters in Table 1. Since each mechanism has a unique density curve, this result indicates that it is possible to distinguish three mechanisms using the time-dependent birth-death model. Moreover, we employed the Akaike Information Criterion (AIC) [56] to evaluate the relative quality of the time-dependent models for nonfunctionalization, neofunctionalization, and subfunctionalization. The data sets simulated from the time-dependent model were used as input data to calculate AIC for nonfunctionalization, neofunctionalization, and subfunctionalization. For each simulated data set, the mechanism with the lowest AIC score was selected and compared with the true mechanism from which the data sets were generated. We reported the percentage of the simulated data sets successfully identifying the true mechanism (Fig. 1c). The overall average of the percentages of samples recovering the true mechanism is about 80 % (Fig. 1c). In addition, subfunctionalization appears to be more difficult than neofunctionalization to distinguish from nonfunctionalization in this modeling framework (Fig. 1c).

**Fig. 2:**
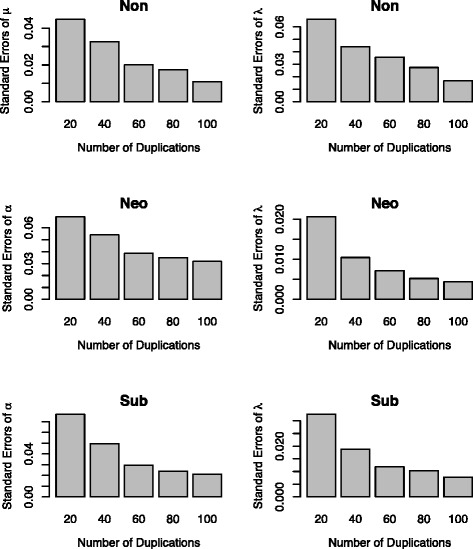
The standard errors of the maximum likelihood estimates of parameters in the age-dependent models for nonfunctionalization, neofunctionliazation, and subfunctionalization

To examine the performance of maximum likelihood estimation, we use the simulated duplication times as data to estimate model parameters. The sample size (the number of duplication times) ranges from 20 to 100. The maximum likelihood estimates of parameters were obtained using Metropolis-Hastings Markov Chain Monte Carlo algorithm. The standard errors of the maximum likelihood estimates are displayed in Fig. 2. For nonfunctionalization, the standard errors of the estimates of *μ* and *λ* decrease as the number of duplication times increases from 20 to 100. Similarly, the standard errors of the estimates of parameters for subfunctionalization and neofunctionalization decrease as the number of duplications grows. However, the estimation of parameter *α* for neofunctionalization does not improve well with the increased number of gene copies (Fig. 2), because duplication times in the simulated data are highly correlated and the auto-correlation between two adjacent duplication times increases as the number of duplication times increases. As a result, when the number of highly correlated duplication times reaches a certain number, adding even more duplication times does not contribute more information for accurately estimating model parameters, especially for neofunctionalization where the loss rate quickly declines to a very low level. Similar results about biases and parameter estimates under constant and time-dependent birth-death processes have been obtained in [57]. Nevertheless, these results suggest that maximum likelihood methods can accurately estimate parameters in the time-dependent birth-death model when the sample size is large.

### Simulation for the age-dependent birth-death model

**Fig. 3:**
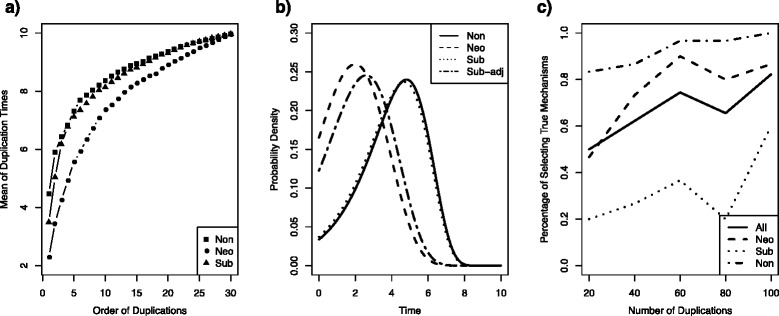
Simulation results of the age-dependent model: (**a**) the means of duplication times simulated with 30 replicates for nonfunctionalization, neofunctionalization, and subfuncitonalization are shown; (**b**) the probability density curves of duplication times for nonfunctionalization, neofunctionalization, and subfunctionalization under the model are shown; (**c**) the percentage of samples identifying the true mechanism with AIC

**Fig. 4:**
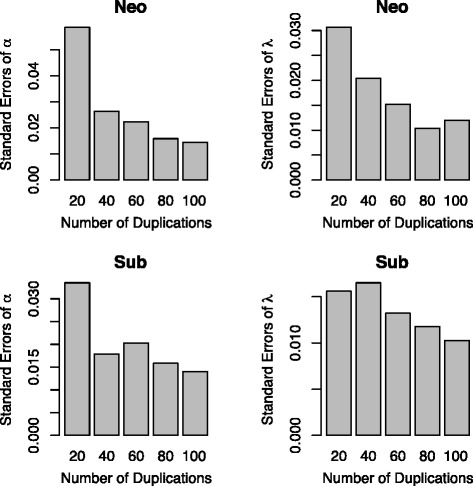
The standard errors of maximum likelihood estimates of parameters in the age-dependent models for neofunctionliazation and subfunctionalization

The simulation for the age-dependent model was conducted with the same parameterization and simulation procedure used for the time-dependent model. We generated duplication times from the age-dependent models for nonfunctionalization, neofunctionalization, and subfunctionalization. The mean duplication times given the current time *T* and gene copy number *n*_*T*_ for the age-dependent models (Fig. 3a) appear to be less dispersed among nonfunctionalization, neofunctionalization, and subfunctionalization than those for the time-dependent models (Fig. 1a). In addition, the density curve for subfunctionalization becomes closer to the nonfunctionalization curve under the age-dependent model (Fig. 3b), compared to the curves for the time-dependent model (Fig. 1b). This is consistent with our expectation, because the age of a gene copy is less than the absolute time *t* and the beginning portion of the concavely declining loss rate of subfunctionalization is similar to the constant rate of nonfunctionalization. In Fig. 3b, the density curve for neofunctionalization is well separated from the density curves for nonfunctionalization and subfunctionalization. In contrast, the loss rate of subfunctionalization is assumed to be a backwards-S-shaped logistic function of time, which is an intermediate state between the loss rates of nonfunctionalization and neofunctionalization. If the loss curve of subfunctionalization moves to the right, it becomes closer to nonfunctionalization (Fig. 3b). Conversely, when the loss rate curve moves to the left, it gets closer to neofunctionalization (Fig. 3d). Although subfunctionalization is an intermediate state between nonfunctionalization and neofunctionalization, it is expected to be more similar to neofunctionalization, which can be tested in real data analysis. The ultimate similarity comes with increasing time, as both neofunctionalization and subfunctionalization culminate in reduced hazard rates, unlike nonfunctionalization. With a fixed duplication rate, these processes are expected to result in an increased number of copies. Conditional on the number of copies, subfunctionalization and neofunctionalization would be consistent with a reduced duplication rate and older duplication times. The overall percentage of samples identifying the true mechanism increases as the number of gene copies grows (Fig. 3c). The percentages of nonfunctionalization and neofunctionalization are significantly higher than the overall percentage. Although the performance of subfunctionalization is below average, the percentage of samples successfully identifying the true subfunctionalization increases to 60 % when the number of gene copies reaches 100. Moreover, the standard errors of the estimates of parameters in the age-dependent model appear to decrease as the number of gene copies grows, suggesting that maximum likelihood methods can accurately estimate parameters in the age-dependent model, when the sample size is large (Fig. 4).

## Discussion

### Summary of the gene family evolution model

We have derived the probability density function for the age-dependent birth-death model, in which the loss rate is a function of the ages of gene copies. In addition, the conditional density function and a joint density function of duplication times with age-dependent loss rate have been developed in above age-dependent model, given the current time *T* and the number of gene copies at the time *T*. The conditional density function is used to efficiently simulate duplication times, and the simulation results suggest that maximum likelihood methods can accurately estimate model parameters in both time-dependent and age-dependent models. In addition, the relative quality of various birth-death models was assessed with AIC. Both time-dependent and age-dependent models can distinguish the three mechanisms (nonfunctionalization, neofunctionalization, and subfunctionalization) with high probabilities when the sample size is large. These results indicate that the probabilistic models derived from the birth-death process with a time-dependent and age-dependent loss rates are useful for understanding the duplication and loss mechanisms of gene families that evolve over time in a single population with caveats discussed.

### Limitations and future study

As duplication times are often not observable, it is desirable to generalize the current model to DNA sequence data. We are currently working along this line to build a generalized model that includes two stochastic processes. The birth and death process is used to derive the probability distribution of a gene family tree, while the mutation process is used to derive the probability distribution of DNA sequence data given the gene family tree. With this generalized model, we can estimate model parameters (duplication and loss rates) from DNA sequence data.

One of the limits of the current model is that it considers gene family evolution in a single population. This model cannot be applied as currently implemented to understand the evolutionary process of gene families from multiple species. To overcome this limit, the current model will be extended in the context of species trees, in which duplication process occurs along the lineages of species trees. This generalization will certainly involve intensive computation, but such a model is quite useful for understanding gene family evolution in the context of the evolution of species. Another limitation of the current age-dependent model is that the likelihood is conditioned on observed extant duplicate copies and does not consider the full generative process including duplicates that were lost before the present. Future work will examine this in the context of Approximate Bayesian Computation [58]. Further, the current model exists in the classes of interspecific models that treat all observations from a single individual from a species as fixed relative to observations from single individuals from other species. Recently, a correction for the effects of population dynamics has been introduced and can be considered in modeling efforts [9]. Missing data and genome assembly error are also not specifically addressed in the modeling framework and their impact on inference also needs to be addressed.

The gene loss models and their interpretations (the relationship between the best fit curve shape and the underlying biology) make assumptions about the relationship between the accumulation of synonymous changes and of non-synonymous changes whereas there is information in the evolution of dN/dS vs. dS that can be taken advantage of in alternative formulations of the likelihood (see [18]). Lastly, the models can be used to make predictions about functional evolution in the absence of actual functional data. While such data does not currently exist in large scale, the future may bring data on the expression levels of protein duplicates compared to an ancestral state as well as binding and enzyme specificities (and enzyme kinetics), all of which can be integrated into a phylogenetic framework. However, even with future comparative proteomic data, one still needs models that treat signals associated with selective pressures (like the models presented here), as neutral changes in expression and functional properties would not lead to changes in retention profiles (the gene loss hazard/survival model) and meaningful lineage-specific biology (see [59] for a discussion of the interplay between molecular phenotypes and biological function in an evolutionary context).

The model as currently developed also assumes that all duplicates in a gene family evolve under the same process. A future opportunity is in examination of large gene family databases like Ensembl [60], HOGENOM [61], or TAED [62], a mixture model of duplicate processes can be applied across all gene families and duplication events to enable a posteriori probabilistic identification of duplication retention mechanisms for individual gene duplication events. The work presented in this manuscript, with a birth-death model in a phylogenetic context, brings this scale of modeling one step closer.

## Conclusions

We develop a generalized birth-death model for probabilistic inference of the evolutionary mechanism for duplicate gene retention based upon the average rate of loss over time of the duplicate. The time-dependent birth-death model assumes a molecular clock that starts ticking for all duplicates at the root. The time-dependent model is then extended to the age-dependent model, which allows the gene loss rate dependent of duplication events. Simulation results indicate that the mechanisms of gene retentions (nonfuncitonalization, neofunctionalization, and subfunctionalization) produce distinct likelihood functions, which can be used with comparative genomic data to quantitatively distinguish those mechanisms.

## Availability of supporting data

This study of a theoretical nature has not generated any novel supporting data.

## Abbreviations

BD: birth-death
MLE: maximum likelihood estimate
